# Optical Properties, Microstructure, and Phase Fraction of Multi-Layered Monolithic Zirconia with and without Yttria-Gradient

**DOI:** 10.3390/ma16010041

**Published:** 2022-12-21

**Authors:** Mi-Hyang Cho, Hyo-Joung Seol

**Affiliations:** 1 Department of Dental Lab, Wonkwang Health Science University, Iksan-si 54538, Republic of Korea; 2 Department of Dental Materials, Dental and Life Science Institute, School of Dentistry, Pusan National University, Yangsan-si 50612, Republic of Korea

**Keywords:** multi-layered zirconia, yttria-gradient, translucency, opalescence, phase fraction

## Abstract

The differences in the optical properties of multi-layered zirconia with and without yttria-gradient are not fully understood. This study aimed to evaluate and compare the optical properties, related microstructures, and phase fractions of multi-layered zirconia with and without yttria-gradient. For this, multi-layered zirconia of 5 mol% yttria (5Y) stabilized (Katana STML) and 4Y/5Y stabilized (e.max MT Multi) were cut layerwise, sintered, and analyzed using the opalescence parameter (OP), average transmittance (AT%), translucency parameter (TP), and contrast ratio (CR). The average grain size and phase fractions were obtained from field-emission scanning electron micrographs and X-ray diffraction patterns, respectively. Although the TP values of Katana STML and e.max MT Multi did not show a significant difference (except for transition layer 1), the results of AT and CR showed that the translucency of e.max MT Multi was slightly higher than that of Katana STML (*p* < 0.05). The opalescence gradient was higher in Katana STML than in the e.max MT Multi. In both zirconia types, translucency increased from the dentin to enamel layer based on the AT, TP, and CR results, while OP decreased (*p* < 0.05). The higher translucency from the dentin to enamel layer in Katana STML was caused by the pigmentation gradient, while in e.max MT Multi, it was caused by the difference in phase fraction and the pigmentation gradient.

## 1. Introduction

Recently, multi-layered zirconia with improved translucency has been widely used in dental prosthetics [1,2]. This multi-layered zirconia is highly esthetic because it expresses the optical properties of natural teeth [3]. Multi-layered zirconia has a dentin (body) layer at the bottom and an enamel (incisal) layer at the top, with one or two transition layers between them [1,2]. Multi-layered zirconia is generally used for anterior prostheses that require esthetics and for the upper structures of post crowns or implant abutments. The method of expressing the translucency gradient in multi-layered zirconia differs depending on the manufacturer, showing that the zirconia in which the content of yttria is different for each layer, and the zirconia in which the same amounts of yttria are contained in each layer [1,4]. In zirconia with the same yttria content in each layer, the pigmentation gradient is attributed to the translucency gradient [2]. In zirconia with a yttria gradient, the yttria content increases from the dentin layer (DL) to the enamel layer (EL), resulting in the EL being the most translucent layer [4].

Early dental zirconia (3Y-TZP) exhibited optically opaque properties that required feldspathic porcelain veneers with high translucency; however, chipping of veneered ceramics was common [5,6,7]. To overcome this problem, monolithic dental prostheses using zirconia have been developed. In yttria-stabilized zirconia, the tetragonal phase that induces the birefringence of light decreases with increasing content of yttria, while the optically transparent cubic phase increases [8,9]. Therefore, zirconia with an increased yttria content was used to improve the translucency of zirconia. In the cubic phase of zirconia, the refractive index is isotropic in all crystallographic directions, whereas the refractive index of the tetragonal phase is anisotropic [10]. In addition, because the cubic phase has a relatively large particle size compared to the tetragonal phase, the grain boundary per unit volume is reduced, attributing to the translucency [11]. However, unlike the tetragonal phase, the cubic phase does not have a stress-induced phase transition function, resulting in relatively poor mechanical properties [11,12,13,14]. Therefore, multi-layered zirconia with a yttria gradient requires a balance between mechanical and optical properties. Commercially available yttria-gradient multi-layered zirconia (3Y-TZP/5Y-TZP) has been reported to have a good balance between mechanical and optical properties [4].

Studies on the optical properties of zirconia, particularly those related to translucency, have been reported [15,16,17,18,19]. Translucency can be assessed by measuring a material’s light transmittance, translucency parameter (TP), and contrast ratio (CR). Transmittance is the amount of light transmitted at a specific wavelength and is expressed as the ratio of the amount of light before and after transmission [20]. The TP represents the color difference between a material of uniform thickness on white and black backgrounds [20,21]. The CR indicates the ratio of a material’s reflectance on a black background to that on a white background of known reflectance. The CR decreases with increasing translucency in dental ceramics [22,23]; thus, the higher the yttria content, the lower the CR value [24]. In a study of 3, 4, and 5 mol% yttria stabilized (3Y, 4Y, 5Y) multi-layered zirconias without yttria-gradient, the TP value of 3Y-zirconia was lower than the TP value of 4-5Y-zirconia. However, there was no significant difference in the TP between 4Y- and 5Y-zirconia [1]. Moreover, in each 3-5Y-zirconia, there was no significant difference in the TP between the DL and EL [1]. On the other hand, when translucency was evaluated using light transmittance, light transmittance increased from the DL to the EL in multi-layered 3-4Y-zirconia without yttria-gradient [2,4]. This tendency was also found in zirconia with yttria-gradient (3Y-TZP in the DL/5Y-TZP in the EL); there was a difference in transmittance for each layer, and the EL showed higher transmittance than the transition layer (TL) and the DL [4].

In addition to translucency, natural tooth enamel exhibits opalescent optical properties [25,26]. Opalescence is an optical phenomenon that allows light scattering at shorter wavelengths in the visible spectrum, resulting in a bluish appearance under reflected light and an orange/brown under transmitted light [20,25]. When zirconia is endowed with opal properties, the prosthesis can respond to light like a natural tooth, increasing vitality [25]. A study on the optical properties of colorless 3Y- and colored 4Y-zirconia of non-multi-layered showed that the AT and TP decreased exponentially with increasing thickness, while the opalescence parameter (OP) increased parabolically with increasing thickness [27,28]. The OP increases when zirconia has a dispersed internal phase. The two-phase nanocomposite of Ceria-stabilized TZP and Al_2_O_3_ showed lower AT and TP and higher OP compared to 3Y-TZP with a single-phase structure [20]. This effect was primarily caused by the significant difference in the refractive index between the two phases of Ceria-stabilized TZP and Al_2_O_3_ [20]. There is no dispersed internal phase in most commercially available monolithic zirconia with multiple layers. However, it is not easy to evaluate their optical properties because there is a gradient in color and translucency from the DL to the EL. In particular, the differences in the optical properties of multi-layered zirconia with and without different yttria contents in each layer are not fully understood. Therefore, the rationale of this study was to provide research data for the development of esthetic zirconia that can mimic the optical properties of natural human teeth by investigating the optical properties of dental multi-layered zirconia with and without yttria-gradient. This study aimed to evaluate and compare the optical properties of multi-layered zirconia with yttria-gradient (4Y/5Y) with those of the zirconia having equal yttria content (5Y) in each layer, as well as the related microstructure and phase fraction. The null hypothesis was that “there is no difference in the optical properties, microstructure, and phase fraction between the two types of multi-layered zirconia”.

## 2. Material and Methods 

### 2.1. Sample Preparation

**Figure 1:**
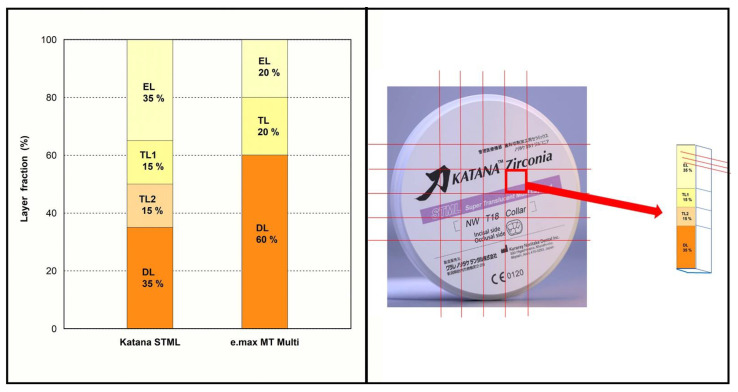
**Left**: layer fraction of used materials (EL: enamel layer; TL: transition layer; DL: dentin layer). **Right**: details of layer-by-layer cut for Katana STML (same method for e.max MT Multi).

Two types of multi-layered zirconia (shade A2) were used (Table 1). One had a yttria-gradient among layers (IPS e.max ZIRCAD MT Multi; IvoclarVivadent, Schaan, Liechtenstein), whereas the other had the same yttria content for each layer (Katana STML; Kuraray Noritake Dental, Tokyo, Japan). The two types of zirconia were cut layer-by-layer according to the fraction of each layer (EL: enamel layer; TL: transition layer; DL: dentin layer) as specified by the manufacturer (Figure 1). A cutter equipped with a diamond wheel (Accutom-100; Struers Company, Copenhagen, Denmark) was used. Zirconia was first cut into elongated cubes of ~12.5 mm (width) × ~12.5 mm (length), as shown in Figure 1. Following this, guidelines were drawn on the elongated cube according to the fraction of each layer, and the inside of each region was cut into plate-shaped specimens with a thickness of ~1.5–2 mm. To control the quality of layer-by-layer cuts, the boundary between layers was not used as a specimen. The specimens were then polished sequentially using 800-, 1200-, 2000-, 3000-, and 5000-grit SiC abrasive papers and measured with a micrometer to obtain a specimen with a thickness of 1.25 mm. The two types of zirconia were then sintered in a sintering furnace (inLab Profire, Dentsply Sirona, Charlotte, NC, USA) using the sintering program supplied by the manufacturer (Table 2). The sintered specimen showed shrinkage of ~20%, and the final size of the sintered specimen was 10.0 mm (width) × 10.0 mm (length) × 1.01 mm (thickness, ± 0.01 mm).

### 2.2. Optical Properties Evaluation

The spectral transmittance and spectral reflectance were measured three times at 10 nm intervals from 360 to 740 nm using a spectrophotometer (CM-3600d, Konica Minolta Sensing, Osaka, Japan), the Spectra-Magic software (version 2.02, Konica Minolta Sensing, Osaka, Japan), a CIE standard D65 light source, and a 2° standard observer (*n* = 4/group). The specimen dimension for optical properties was 10.0 mm (width) × 10.0 mm (length) × 1.01 mm (thickness, ± 0.01 mm). The total light transmittance was obtained by placing the specimen in the inlet hole of the integrating sphere in transmission mode. This was divided by the transmittance measured with no specimen to obtain a percentage of light transmittance between 0% (opaque) and 100% (transparent). The average transmittance (AT) was obtained by dividing the sum of transmittance (%) at each wavelength by the number of data points [20,29].

For spectral reflectance measurements, the specimen was placed on white (CIE L * = 99.07, a * = −0.09, b * = 0.97) and black (CIE L * = 9.07, a * = 0.71, b * = −0.41) backgrounds. A software (Spectra-Magic, Version 2.02, Konica Minolta Sensing, Osaka, Japan) was used to determine the color coordinates as follows: L * (brightness), a * (red–green chromaticity index), b * (yellow–blue chromaticity index), and Y (tristimulus value). The TP was calculated using the following equation:

TP = [(L *_W_ − L *_B_)^2^ + (a *_W_ − a *_B_)^2^ + (b *_W_ − b *_B_)^2^]^1/2^ (1)

where subscripts W and B denote the color coordinates measured on white and black backgrounds, respectively [21,30,31].

To obtain the CR of the specimen, the spectral reflectance (Y) of the specimen obtained on the black (Yb) and white (Yw) backgrounds were put in the equation CR = Yb/Yw [32]. The contrast ratio of “0” was considered transparent and “1” opaque [32,33,34].

The OP was determined according to the following equation [20,35]:

OP = [(a *_T_ − a *_R_)^2^ + (b *_T_ − b *_R_)^2^]^1/2^ (2)

where the subscript T denotes the transmitted lights, and the subscript R denotes the reflected lights measured on a black background.

### 2.3. Field Emission Scanning Electron Microscopy (FE-SEM) Analysis

To observe the specimen’s microstructure, it was sputter-coated with platinum for 60 s and then subjected to FE-SEM analysis (JSM-7200F, Jeol, Akishima, Japan) at an acceleration voltage of 10 kV (*n* = 1/group). The line interception method was used to measure the grain size [36]. More than 800 grains were measured from 4 FE-SEM images per layer using the Image J Software (version 1.53e, National Institute of Health, Bethesda, Rockville, MD, USA). Average particle size (D) was calculated using the following Equation (1):

(3) $$D=1.56\;\frac{C}{MN}$$

where *C* is the length of the test line, *M* is the magnification of the micrograph, and *n* is the number of intercepts. In this equation, 1.56 was used as a correction factor [1,36].

### 2.4. X-ray Diffraction (XRD) Analysis

For the specimen’s XRD analysis, high-resolution XRD (X’Pert3 Powder; PANalytical, Amsterdam, The Netherlands) was used (*n* = 1/group). The measurements were performed at a tube voltage of 40 kV, tube current of 30 mA, and step size of 0.013° in the range of 20–80° (2θ). In addition, Cu-Kα radiation filtered with Ni was used. Topas Academic software V 7.20 (Bruker AXS, Karlsruhe, Germany) was used to perform Rietveld analysis of the obtained X-ray diffraction patterns. Relative phase fraction and lattice parameters were analyzed for the monoclinic (space group: P2_1_/c), tetragonal (space group: P4_2_/nmc), and cubic (space group: Fm3m) phases. The Y_2_O_3_ content (mol%) in the tetragonal phase was calculated based on the lattice parameters (*a*,*c*) using Equations (4) and (5) [37,38]:

(4) $${\mathrm{YO}}_{1.5}\;(\mathrm{mol}\%)=\frac{1.0223-c/a\sqrt{2}}{0.001319}$$

Y_2_O_3_ (mol%) = 100 × YO_1.5/_(200 − YO_1.5_) (5)

### 2.5. Statistical Analysis 

The experimental results were analyzed at a significance level of 0.05 using the statistical program SPSS 25.0 (Statistical Product and Service Solutions 25.0, IBM Co., Armonk, NY, USA). The Shapiro–Wilk test was performed to analyze the normality of the data. Data for the AT, TP, CR, and OP were analyzed using two-way ANOVA, post-hoc Tukey’s HSD test, and Student’s *t*-test. Grain-size data were analyzed using a generalized linear model, as the data did not satisfy normality.

## 3. Results

### 3.1. Total Transmittance 

**Figure 2:**
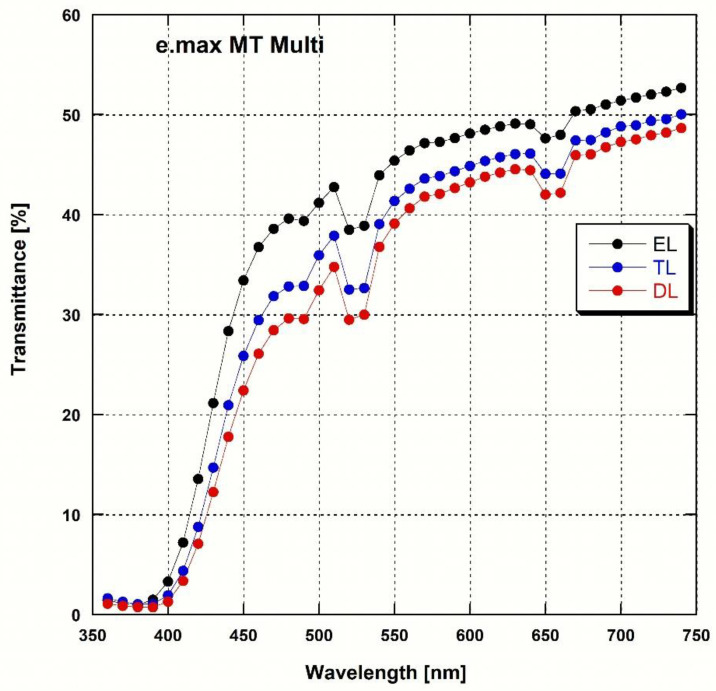
Spectral transmittance curves for each layer of the 4Y/5Y-zirconia (e.max MT Multi).

**Figure 3:**
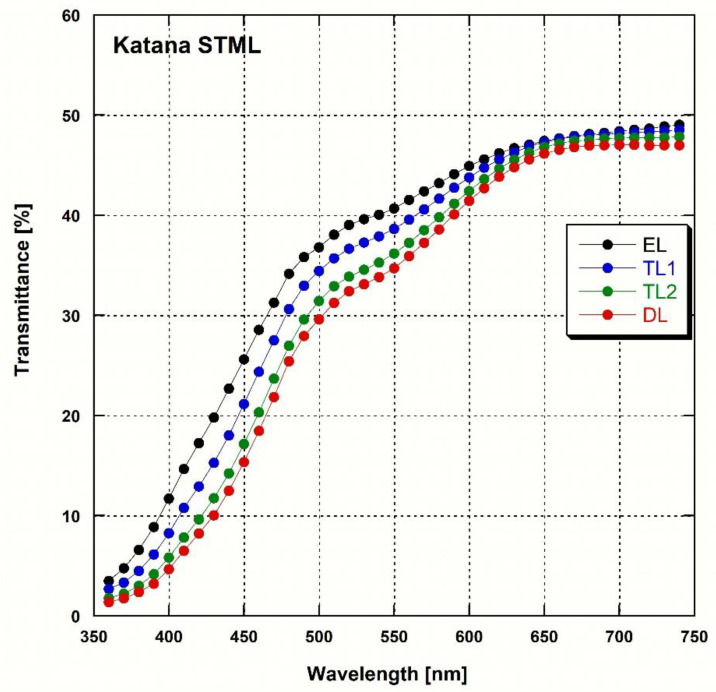
Spectral transmittance curves for each layer of the 5Y-zirconia (Katana STML).

The total transmittance of the two types of zirconia at each wavelength (Figure 2 and Figure 3) showed that the transmittance increased with the wavelength in both cases. The reduction in the peaks at 520 and 650 nm was observed only in e.max MT Multi and not in Katana STML. The two-way ANOVA showed that the zirconia type (*p* < 0.001), layer (*p* < 0.001), and the interaction between zirconia type and layer (*p* = 0.002) influenced AT. As a result of the post-hoc analysis (Table 3), both zirconias showed higher AT from the DL to the EL (*p* < 0.05). The e.max MT Multi showed a statistically higher AT (*p* < 0.05) than Katana STML in all layers with the exception of transition layer (TL) 1. 

### 3.2. Translucency Parameter (TP)

The two-way ANOVA revealed that the zirconia type (*p* < 0.001) and layer (*p* < 0.001) influenced the TP. As a result of the post-hoc analysis (Table 3), both zirconia types showed a tendency to increase in the TP from the DL to the EL (*p* < 0.05). There was no statistically significant difference in the TP values between the two zirconia layers (*p* > 0.05, except for TL1).

### 3.3. Contrast Ratio (CR)

The two-way ANOVA revealed that zirconia type (*p* < 0.001), layer (*p* < 0.001), and the interaction between zirconia type and layer (*p* < 0.001) influenced the CR. The post-hoc analysis (Table 3) showed that the CR decreased from the DL to the EL in both zirconia types (*p* < 0.05). The CR of Katana STML was higher than that of e.max MT Multi in all layers (*p* < 0.05).

### 3.4. Opalescence Parameter (OP) 

The two-way ANOVA revealed that zirconia type (*p* < 0.001), layer (*p* < 0.001), and the interaction between zirconia type and layer (*p* = 0.002) influenced the OP. The OP decreased from the DL to the EL in both types of zirconia (Table 3, *p* < 0.05). There was no significant difference in the OP values for the EL between Katana STML and e.max MT Multi; however, for the DL, Katana STML had a higher OP than that of e.max MT Multi (*p* < 0.05). The difference in the OP values between the EL and the DL was more significant for Katana STML than for e.max MT Multi.

### 3.5. FE-SEM Analysis

**Figure 4:**
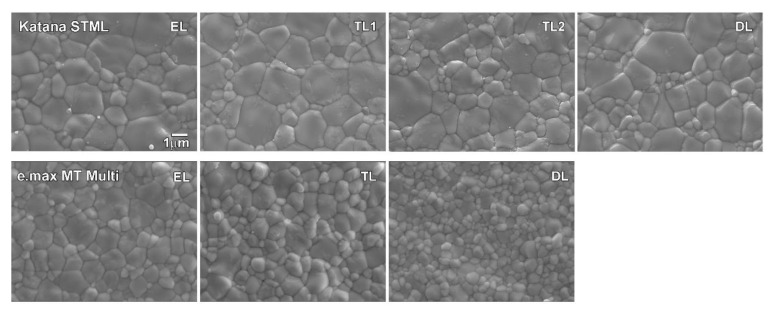
Microstructure for each layer of sintered zirconia.

Both types of zirconia showed an equiaxed crystal structure in the FE-SEM image (Figure 4). All layers of Katana STML and all layers (except the DL) of e.max MT Multi had nonuniform grain sizes, and this trend was more prominent in Katana STML. Statistical analysis revealed that zirconia type (*p* < 0.001), layer (*p* < 0.001), and the interaction between zirconia type and layer (*p* < 0.001) affected the average grain size. As shown in Table 4, the average grain size of e.max MT Multi increases from the DL to the EL (*p* < 0.05). For Katana STML, there was no significant difference in the average grain size in each layer (*p* > 0.05). Katana STML had a larger average grain size than e.max MT Multi in all layers (*p* < 0.05).

### 3.6. XRD Analysis 

**Figure 5:**
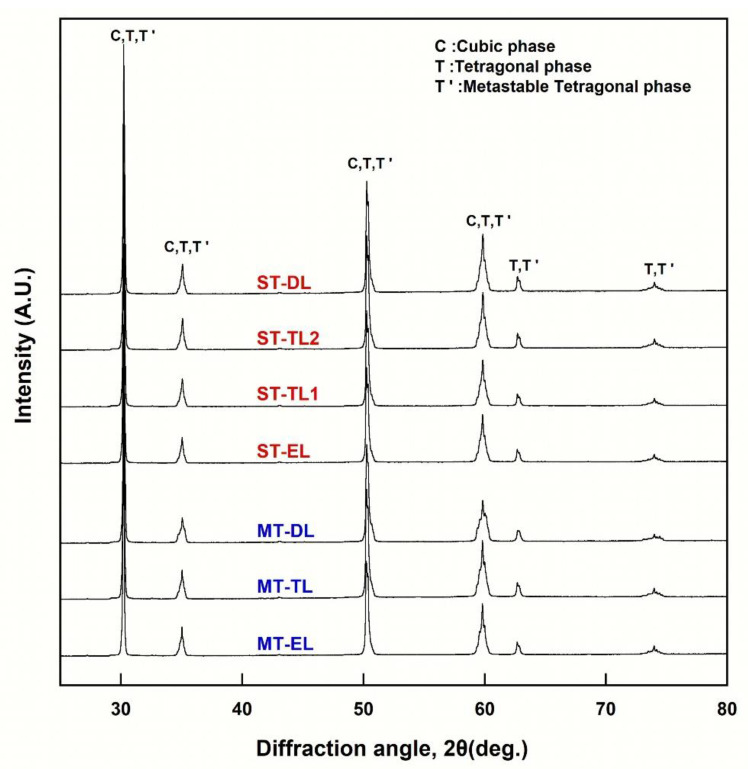
XRD patterns for each layer of sintered zirconia (ST: Katana STML; MT: e.max MT Multi).

Rietveld analysis of the XRD patterns (Figure 5, Table 5) showed that both types of zirconia primarily consisted of three phases: cubic (C), tetragonal (T), and metastable tetragonal (T′) in all layers, and the monoclinic (M) phase content was less than 0.06 wt%. In Katana STML, the phase fraction of all layers was similar, with a C-phase of ~57–58 wt%, T’-phase of ~27–29 wt%, and T-phase of ~14–15 wt%. In the e.max MT Multi, the phase fraction of each layer was different. The phase fractions of the EL for e.max MT Multi were ~50 wt% for the C-phase, 37.5 wt% for the T′-phase, and ~13 wt% for the T-phase. The fraction of the T-phase increased from the EL to the DL, whereas the T′- and C-phases decreased. In the DL of e.max MT Multi, the C-phase content was ~47 wt%, ~29 wt% for the T′-phase, and ~24 wt% for the T-phase.

The axial ratio (*c*/*a*√2, tetragonality) and yttria content of each tetragonal phase (T-phase, T′-phase) were similar, regardless of the zirconia type and layer. The axial ratios (*c*/*a*√2) were ~1.016 for the T-phase and ~1.005 for the T′-phase. The yttria content was ~2.5 mol% in the T-phase and ~6.9 mol% in the T′-phase.

## 4. Discussion

This study showed differences in the optical properties, microstructure, and phase fraction of two types of multi-layered zirconia, against the null hypothesis. The Katana STML and e.max MT Multi used in this experiment were multi-layered zirconia, and both had a yttria content of 5 mol% in the EL. However, Katana STML had little difference in yttria content for each layer [1], whereas e.max MT Multi had a yttria-gradient. 

Measuring the light transmittance at each wavelength showed a reduction in the peaks at 520 and 650 nm only in e.max MT Multi but not in Katana STML. This difference was owing to the difference in the type and content of the added pigments [2,27]. In both zirconias, the AT and TP increased from the DL to the EL, while the CR decreased (*p* < 0.05). These results indicate that translucency increases from the DL to the EL. This tendency was also reported for multi-layered zirconia with lower yttria content [2]. The AT (%) of Katana STML was ~35 in the EL and ~31 in the DL, which is consistent with values reported in a previous study [4]. The e.max MT Multi showed slightly higher AT (%) values for each layer than the Katana STML (except for TL1, *p* < 0.05). The CR was lower in e.max MT Multi than in Katana STML (*p* < 0.05). Because translucency and the CR are inversely proportional, the results of the AT and CR showed that the translucency of e.max MT Multi was slightly higher than that of Katana STML. However, with the exception of TL1, the TP values of Katana STML and e.max MT Multi were not significantly different. In this study, the ELs of the two zirconia exhibited a TP value of ~13, which was slightly higher than the TP value reported without dividing the zirconia sample by layer (14.3 for Katana STML, 14.2 for e.max MT Multi) [39]. This may be caused by the fact that additional mirror polishing was performed after sintering the zirconia samples. 

Human tooth enamel is opalescent, appearing bluish in reflected color and orange/brown in transmitted color [26]. In this study, the OP of zirconia was obtained using a comparison of the colors measured in reflectance and transmittance modes. There was no statistically significant difference between the OP values of Katana STML and e.max MT Multi for the EL and TL1. The OP value of the EL was ~15 in this study, which was lower than that of the human tooth enamel (19.8–27.6) [25]. A lower OP value has also been reported for the composite resin or core/veneer ceramics; however, the value differs depending on the type of material and the presence of distributed particles inside [40,41]. In contrast, Kanata STML (~20) showed a higher OP value than e.max MT Multi (~18) (*p* < 0.05) for the DL. Visual inspection of the two types of zirconia (A2 shade) revealed that the color gradient of Kanata STML was more prominent than that of e.max MT Multi. This may be related to the higher opalescence gradient in Katana STML than in e.max MT Multi. In this study, translucency increased from the DL to the EL based on the AT, TP, and CR results, while the OP decreased (*p* < 0.05) for both types of zirconia. This trend was also reported in a study on the changes in translucency and the OP according to the thicknesses of 3Y- and 4Y-zirconia [27,28]. Similarly, it has been reported that the OP increases while translucency decreases when a finely dispersed internal phase is present in zirconia [20]. 

The cubic phase of zirconia has an isotropic refractive index in all crystallographic directions, whereas it is anisotropic in the tetragonal phase, resulting in birefringence [10]. Owing to birefringence, polycrystalline Y-TZP causes discontinuity in the refractive index at the grain boundaries when adjacent grains do not have the same crystallographic orientation. As a result, both reflection and refraction of light occur at the grain boundary, thereby reducing light transmittance [10]. In the tetragonal phase of 3Y-TZP, if the particle size is increased to reduce the grain boundaries (above 1 μm), a spontaneous phase transformation from tetragonal to monoclinic occurs, thereby reducing the mechanical properties [42]. Therefore, a nanocyrstalline structure with a particle size of less than 100 nm should be used to obtain excellent mechanical properties together with translucency in 3Y-TZP; however, there are considerable technical limitations [10]. As an alternative, some of the tetragonal grains were replaced with cubic grains by adding yttria to the early 3Y-TZP to improve the translucency of zirconia. In this study, commercial zirconia with yttria contents of 5 and 4/5 mol% was used. Rietveld analysis of the XRD patterns (Figure 5, Table 5) showed that the phase fraction of each layer was similar in Katana STML, with ~57–59 wt% C-phase, ~27–29 wt% T′-phase, and ~14–15 wt% T-phase. Because the T′-phase of zirconia has lattice constants very close to those of the C-phase, the peak positions in XRD patterns are close to the peak positions of the C-phase [37,43]. Therefore, it is not easy to distinguish the T’-phase from the C-phase. 

The phase diagram presented by Scott [44] shows that 3Y-zirconia is separated into a C-phase containing high yttrium and a T-phase containing low yttrium at the sintering temperature. However, it has been reported that phase equilibrium does not occur completely under normal sintering conditions, resulting in the formation of the T′-phase (so-called pseudo-cubic) instead of the C-phase in 3Y-zirconia [37]. The majority of previous studies that analyzed the XRD pattern of Katana STML did not differentiate between the T-phase and T′-phase. As a result, the reported phase fraction for Katana STML showed a wide range of values for the T-phase (30–47 wt%) and the C-phase (53–70 wt%) [1,8,9,33]. The fraction of the C-phase obtained in this study was ~57–59 wt%, which was within the range of the values reported in previous studies. Because the T- and T′-phases were differentiated in this study, the fraction of the T-phase was lower than that reported in previous studies. However, the sum of the two types of tetragonal phases (T-phase and T′-phase) was ~42–43 wt%, which is within the range of the values reported in previous studies. The T′-phase has a higher yttria content than the T-phase [37,45], as shown in Table 5. A comparison of the axial ratio (*c*/*a*√2, tetragonality) obtained from the lattice constants of the two types of tetragonal phases shows that the T′-phase has a smaller axial ratio than the T-phase owing to the higher yttria content [28,37,46]. The obtained axial ratio was ~1.016 for the T-phase; however, for the T′-phase, it decreased to ~1.005, which is consistent with values in the literature [37,46]. Owing to the decreased tetragonality, the T′-phase induces less light scattering than the T-phase, which can improve translucency [46]. In the literature, Katana STML showed an increase in grain size and translucency as the sintering temperature increased from 1350 °C to 1600 °C [47]. In this study, all layers of Katana STML were sintered at 1550 °C, and the average grain sizes of all layers were not significantly different from each other (Table 4, *p* > 0.05). Moreover, because the phase fraction was similar in all layers, the translucency gradient in Katana STML must be caused simply by the gradient of pigmentation [2].

Unlike Katana STML, e.max MT Multi has different yttria contents in each layer according to the manufacturer’s information. Rietveld analysis showed that each layer had a different phase fraction in e.max MT Multi. From the EL to the DL, the fraction of the T-phase increased with a simultaneous decrease in the T′- and C-phase fractions. In the EL with the highest yttria content, the fraction of each phase was ~50 wt% for the C-phase, ~37 wt% for the T’-phase, and ~13 wt% for the T-phase. The phase fraction of the TL was closer to that of the EL than to that of the DL. For the DL, which is the bottom layer, the fraction of the C-phase (~47 wt%) was not apparently different from that of the EL, but the fraction of the T′-phase was reduced to ~29 wt%, and that of the T-phase was increased to ~24 wt%. Such a change in the fraction of the T′- and T-phases increases the tetragonality in zirconia, and consequently, the translucency decreases. A study of 3-5Y-zirconia showed that with decreasing yttria content, the T-phase fraction increased as the T′-phase fraction decreased, showing the lowest translucency in 3Y-zirconia [46]. Similarly, sintering 3-5Y-zirconia followed by rapid cooling resulted in a decrease in the T-phase with a simultaneous increase in the T′-phase, which improved the translucency of each zirconia [46]. These results suggest that in addition to the gradient of pigmentation, the difference in phase fraction attributed to higher translucency from the DL to the EL in e.max MT Multi. The grain size analysis showed that the average grain size of e.max MT Multi decreased from the EL to the DL (*p* < 0.05). This was considered to be due to an increase in the T-phase fraction from the EL to the DL. In this study, e.max MT Multi had a smaller average grain size than Katana STML in all the layers (*p* < 0.05). In addition, e.max MT Multi had a lower fraction of the C-phase, which contributes to translucency, than Katana STML in all layers. Nevertheless, the AT and CR results showed that the translucency of e.max MT Multi is slightly higher, possibly due to more pigmentation in Katana STML than in e.max MT Multi. 

By investigating the optical properties of dental multi-layered zirconia, it is possible to develop esthetic zirconia materials that can mimic the optical properties of natural human teeth without the requirement of veneering porcelain. In this study, multi-layered zirconia of A2 shade, which is a widely used shade, was tested. The average TP values for the tested zirconia were lower than the reported values of 1 mm-thick human enamel and dentin [3]. The obtained OP values of the EL were lower than those of human enamel in both zirconia types [25]. A limitation of this study is that only two types of zirconia were used to observe the optical properties, microstructure, and phase fraction, and the test was performed with a specimen of only one thickness (1 mm). Therefore, the results obtained in this study may be different if zirconia is used at various thicknesses by different manufacturers. In this study, the two types of zirconia showed different phase fractions, which may cause differences in various mechanical properties, and further study is required.

## 5. Conclusions

In conclusion, the translucency of e.max MT Multi was slightly higher than that of Katana STML, as assessed using the AT and CR, but was similar in the TP results. The opalescence gradient was higher in Katana STML than in e.max MT Multi. The translucency gradient in Katana STML was caused simply by the gradient of pigmentation. However, in e.max MT Multi, it was caused by the difference in phase fraction and the gradient of pigmentation.

## Figures and Tables

**Table 1:** Chemical composition of the materials used.

Material	Zirconia Type		Chemical Composition (wt%)			
		ZrO_2_	HfO_2_	Y_2_O_3_	Al_2_O_3_	Others
e.max MT Multi	5Y-PSZ (enamel layer) 4Y-PSZ (dentin layer)	86–93.5	≤5	>6.5–≤ 8	≤1	≤1
Katana STML	5Y-PSZ	88–93 (ZrO_2_ + HfO_2_)		7–10	0–2 (Al_2_O_3_ + Others)	

**Table 2:** Sintering protocols for the materials used.

Material	Code	Stage	Heating and Cooling Rate	Temp.	Holding Time (min)
			(°C/min)	(°C)	
e.max MT Multi	MT	1	10	900	30
		2	3	1500	120
		3	−10	900	0
		4	−8	300	0
Katana STML	ST	1	10	1550	120
		2	−10	100	0

**Table 3:** Optical properties of each group of zirconia specimens (mean ± SD).

AT (%)	EL	TL1	TL2	DL
* e.max MT Multi	37.35 ^Bc^ (0.65)	33.55 ^Ab^ (0.64)	33.55 ^Bb^ (0.64)	31.63 ^Ba^ (0.41)
Katana STML	35.48 ^Ad^ (0.42)	33.62 ^Ac^ (0.42)	31.71 ^Ab^ (0.45)	30.59 ^Aa^ (0.21)
**TP**	**EL**	**TL1**	**TL2**	**DL**
* e.max MT Multi	12.89 ^Ab^ (0.20)	12.41 ^Aa^ (0.13)	12.41 ^Aa^ (0.13)	12.06 ^Aa^ (0.32)
Katana STML	13.06 ^Ab^ (0.12)	13.00 ^Bb^ (0.12)	12.57 ^Aa^ (0.19)	12.44 ^Aa^ (0.19)
**CR**	**EL**	**TL1**	**TL2**	**DL**
* e.max MT Multi	0.69 ^Aa^ (0.006)	0.72 ^Ab^ (0.002)	0.72 ^Ab^ (0.002)	0.74 ^Ac^ (0.006)
Katana STML	0.72 ^Ba^ (0.004)	0.73 ^Bb^ (0.001)	0.75 ^Bc^ (0.005)	0.76 ^Bd^ (0.004)
**OP**	**EL**	**TL1**	**TL2**	**DL**
* e.max MT Multi	14.52 ^Aa^ (0.46)	17.41 ^Ab^ (0.84)	17.41 ^Ab^ (0.84)	18.03 ^Ab^ (0.69)
Katana STML	15.23 ^Aa^ (0.46)	17.17 ^Ab^ (0.60)	19.34 ^Bc^ (0.64)	19.82 ^Bc^ (0.49)

* Data for TL were used twice (TL1 and TL2) for comparison with TL1 and TL2 of Katana STML. The same uppercase letter indicates no statistically significant difference between zirconia products (*p* > 0.05), and the same lowercase letter indicates no statistically significant difference between layers (*p* > 0.05).

**Table 4:** Average grain size obtained for each specimen.

Material	Grain Size (μm)	EL	TL1	TL2	DL
* e.max MT Multi	M	1.243 ^Ac^	1.122 ^Ab^	1.122 ^Ab^	0.701 ^Aa^
	±SD	(0.136)	(0.111)	(0.111)	(0.057)
Katana STML	M	1.616 ^Ba^	1.609 ^Ba^	1.596 ^Ba^	1.624 ^Ba^
	±SD	(0.221)	(0.284)	(0.234)	(0.199)

* Data for TL were used twice (TL1 and TL2) for comparison with TL1 and TL2 of Katana STML. The same uppercase letter indicates no statistically significant difference between zirconia products (*p* > 0.05), and the same lowercase letter indicates no statistically significant difference between layers (*p* > 0.05).

**Table 5:** Rietveld analysis results of XRD patterns for each specimen.

Material	e.Max MT Multi			Katana STML			
Parameter	EL	TL	DL	EL	TL1	TL2	DL
Rwp (%)	3.0952	3.0204	2.9335	3.3502	3.3291	3.5352	3.2857
GOF	3.1381	3.3069	3.1238	3.3720	3.3635	3.8684	3.5971
**T-phase**							
Fraction (wt%)	12.74 (29)	16.72 (31)	24.14 (34)	14.83 (29)	14.56 (26)	14.10 (29)	14.26 (28)
*a* (Å)	3.6072	3.6076	3.6076	3.6074	3.6071	3.6078	3.6073
*c* (Å)	5.1814	5.1819	5.1813	5.1828	5.1829	5.1828	5.1829
Tetragonality, *c*/*a*√2	1.0157	1.0157	1.0156	1.0159	1.0160	1.0158	1.0160
Y_2_O_3_ (mol%)	2.5694	2.5752	2.6222	2.4825	2.4411	2.5274	2.4635
**T′-phase**							
Fraction (wt%)	37.51 (53)	35.74 (54)	28.62 (46)	28.25 (44)	26.76 (39)	29.35 (47)	28.74 (44)
*a* (Å)	3.6254	3.6262	3.6262	3.6262	3.6259	3.6264	3.6262
*c* (Å)	5.1544	5.1551	5.1553	5.1558	5.1555	5.1555	5.1554
Tetragonality, *c*/*a*√2	1.0053	1.0052	1.0053	1.0054	1.0054	1.0053	1.0053
Y_2_O_3_ (mol%)	6.8766	6.9136	6.8967	6.8545	6.8438	6.9038	6.8882
**C-phase**							
Fraction (wt%)	49.70 (60)	47.51 (65)	47.24 (61)	56.92 (51)	58.67 (46)	56.55 (57)	57.00 (52)
*c* (Å)	5.1367	5.1377	5.1373	5.1390	5.1389	5.1388	5.1389

Values in parentheses correspond to the estimated standard deviation in the least significant figure to the left; GOF = goodness of fit.

## Data Availability

Not applicable.
